# Biochemical composition and function of subalpine shrubland and meadow soil microbiomes in the Qilian Mountains, Qinghai–Tibetan plateau, China

**DOI:** 10.7717/peerj.13188

**Published:** 2022-04-04

**Authors:** Qiuyun Fan, Yuguo Yang, Yuqing Geng, Youlin Wu, Zhanen Niu

**Affiliations:** 1 School of Forestry, Beijing Forestry University, Beijing, China; 2 Huzhu Tu Autonomous County Beishan Forest Farm, Haidong, Qinghai, China

**Keywords:** Subalpine shrubland, Meadow, Microbial community, Phospholipid fatty acid, Soil enzyme

## Abstract

Microorganisms participate in the soil biogeochemical cycle. Therefore, investigating variations in microbial biomass, composition, and functions can provide a reference for improving soil ecological quality due to the sensitivity of microorganisms to vegetation coverage changes. However, the differences in soil microorganisms between shrubland and meadow have not been investigated in ecologically vulnerable subalpine areas. This study aimed to investigate the biochemical composition and functions of the soil microbial community under two shrublands and a meadow at high altitudes (3,400–3,550 m). Three sites under two shrublands, *Rhododendron thymifolium* (RHO) and *Potentilla fruticosa* (POT), and one meadow dominated by *Kobresia myosuroides* (MEA), were selected on the southern slope of the Qilian Mountains on the northeastern edge of the Qinghai–Tibetan Plateau, China. Soil physicochemical properties, the microbial community composition expressed by the phospholipid fatty acid (PLFA) biomarker, and enzyme activities were analyzed as well as their relationships. The results showed that water holding capacity and the soil carbon, nitrogen, and potassium content in RHO and POT were higher than those in the MEA. Moreover, the soil active carbon, dissolved organic carbon, total nitrogen, and dissolved total nitrogen content in RHO were higher than those in POT. The abundance of total PLFAs, bacteria, and fungi beneath the shrublands was considerably higher than that in the MEA. The PLFA abundance in RHO was significantly higher than that in POT. The fungal-to-bacterial ratio of RHO and POT was significantly higher than that in the MEA. The activities of β-glucosidase, cellobiohydrolase, and leucine aminopeptidase were the highest in RHO among the three vegetation types, followed by POT and MEA. The redundancy analysis indicated that the biochemical composition of the soil microorganisms and enzyme activities were driven by total nitrogen, dissolved organic carbon, water holding capacity, and soil organic carbon. Therefore, shrublands, which have higher biomass, can improve soil moisture status, increase soil carbon and nitrogen content (especially active carbon and active nitrogen), and further increase the abundance of total PLFAs, bacteria, and fungi. The increase of microbial biomass indirectly enhances the activity of relevant soil enzymes. The variations in PLFA abundance and enzyme activities can be attributed to shrub species, especially evergreen shrubs, which create more favorable conditions for soil microorganisms. This study provides a theoretical basis for investigating the soil biogeochemical cycle and a scientific basis for soil management and vegetation restoration in the subalpine regions.

## Introduction

The soil biogeochemical cycle plays a key part in structuring terrestrial ecosystems (Hobara et al., 2014). Soil microorganisms are crucial in transforming soil carbon and nutrients and they serve as a link in nutrient flow between above and underground ecosystems (Kong et al., 2011). Soil enzymes are mainly secreted by soil microorganisms, and their activities can reflect soil microorganism function (Sinsabaugh, Hill & Follstad Shah, 2009). The structure and functions of the soil microbial community can be shaped by plant species *via* litter and root exudates, which, in turn, affect soil processes and plant performance (Lv et al., 2017). Understanding the composition and functions of soil microorganisms beneath different vegetation types can provide insights into soil organic matter decomposition, nutrient mineralization, and other soil ecological processes (Nkongolo & Narendrula-Kotha, 2020; Yokobe, Hyodo & Tokuchi, 2018).

Vegetation type in mountainous regions gradually transitions from forests and shrublands to meadows with increasing elevation. The distribution model between the shrub and meadow generally presents a staggered form due to variations in the terrain (Duniway, Snyder & Herrick, 2010). Meadows are generally located at the very top of the vegetation vertical zone spectrum and play important ecological roles in soil and water conservation, carbon sequestration, and economic development in subalpine regions (Yin et al., 2021). Shrublands with higher aboveground biomass and roots have greater potential to alter soil properties and function than meadows (Li, Hu & Li, 2019). Furthermore, the “fertile island” effect strengthens the stress resistance and growth of shrubs, which helps plants to adapt better to site conditions (Zhao & Liu, 2013). Furthermore, the change in vegetation type strongly influences soil microbial communities (Li et al., 2017).

With the encroachment of herbs by shrub plants attracting increasing attention, the shifts in soil bacterial and fungal communities caused by shrubs have been recognized (Rusterholz, Schneuwly & Baur, 2018). A richer soil microorganism composition and more exclusive operational taxonomic units occur in the soil under shrubs than in soil under grasses (Lan et al., 2021). However, Qiang et al. (2021) reported that the bacterial and fungal community structures in the grassland and shrubland were relatively similar. Interestingly, a mild-to-moderate transformation of herbs to shrublands have been observed to increase total soil phospholipid fatty acids (PLFAs) (Jin et al., 2019). Furthermore, the abundance of gram-positive bacteria can be greater in shrublands than in grasslands (Zhao et al., 2019). Moreover, soil enzyme activity was higher in cold, wet mountains than in hot, dry valleys and the valley-mountain ecotone (Cao et al., 2021). Furthermore, higher hydrolytic enzyme activity was observed in subalpine soils than in alpine soils, and an upwards vegetation shift may alter the soil enzymatic activities (D’Alò et al., 2021). Notably, the shift of individual soil enzyme activity was inconsistent, as the activities of some soil enzymes decreased from 3,200 to 3,600 m and increased from 3,800 to 4,000 m, whereas others showed similar fluctuations with increasing altitude in subalpine regions (Fan et al., 2021). In addition, a high degree of spatial variation of soil fungal diversity and community composition substantially exceeded the effects of the vegetation shift (Collins et al., 2018). Therefore, the uncertain effect of vegetation variation on soil microorganisms suggests a need for further research on the relationship between shrub-dominated communities and meadows in colder and higher subalpine regions.

The Qilian Mountains, on the northeastern edge of the Qinghai–Tibetan Plateau, play a significant role in retaining water, mitigating climate change, and maintaining biodiversity (Qian et al., 2019). Shrublands, comprising evergreen shrubs and alpine deciduous shrubs, and meadows are the primary vegetation type in high elevation regions (Qi et al., 2021). A combination of environmental factors and anthropogenic activities have substantially reduced shrubland area since the 1950s (Sun et al., 2016). The Chinese government has launched the Natural Forest Resource Protection Project to protect forestland resources and improve ecosystem service functions (Wang et al., 2020). Over the past 20 years, shrub cover has increased and dominated certain meadows in the Qilian Mountains (Xiang et al., 2019; Du et al., 2021). Previous studies investigating the ecological effect of vegetation variation have focused on plant distribution, soil carbon pools, and soil nutrient cycles (Qin et al., 2019; Wan et al., 2019; Qin et al., 2016). PLFAs are stable components of microbial cell structures. Therefore, analyzing the types and proportions of PLFAs can reveal the diversity of soil microbial structures (Frostegård, Tunlid & Bååth, 2011). Analysis of the PLFAs of soil microorganisms has been widely used due to its sensitivity in reflecting microbial community structure and reproducibility (Francisco et al., 2016; Fanin et al., 2019). Additionally, the fungal-to-bacterial (F:B) ratio is a useful indicator of relative carbon availability and the extent of energy limitation for the soil microorganism community (Bailey, Smith & Bolton, 2002). However, variations in the microbial community characteristics between shrub and meadow plant species in subalpine regions in the Qilian Mountains have received little attention from researchers. The objectives of this study were to investigate (1) the variations in the biochemical composition of soil microorganisms and functions represented by soil enzyme activity in shrub and meadow environments and (2) the major soil physicochemical properties driving variation in the soil microbial biochemical composition and soil enzyme activity. We aimed to identify potential mechanisms for soil ecosystem restoration in the subalpine region and to provide a scientific basis for soil management and vegetation restoration in the Qilian Mountains.

## Materials and Methods

### Study area

The study area is located within the Beishan Forest Farm (36°42′–37°06′ N, 102°06′–102°43′ E) of Tu Autonomous County of Huzhu in the middle and lower reaches of the Datong River, the southern slope of the Qilian Mountains, Qinghai Province. The topography slopes downward from 4,308 m in the northwest to 2,100 m in the southeast. This area is characterized by a cold semi-humid plateau climate with a long winter. The annual mean temperature is 0–3.8 °C, with a mean daily temperature of −14.5 °C in January and a maximum of 15.7 °C in July. The average annual precipitation is 470–680 mm, with a mean annual evaporation of 1,194 mm.

The study area is a shrub–meadow ecotone located at an elevation of approximately 3,200–3,700 m in the subalpine region, with shrubs and meadows distributed in patches. The main shrub species are *Rhododendron thymifolium* Maxim., *Salix oritrepha Schneid*., *Potentilla fruticosa* L., *Potentilla glabra* Lodd., *Rhododendron przewalskii* Maxim., and *Caragana jubata* (Pall.) Poir. The main herb species in the meadows are *Stellaria media* (L.) Vill., *Elymus dahuricus* Turcz., *Elymus nutans* Griseb., *Kobresia myosuroides*, *Kobresia pygmaea* C. B. Clarke, *Kobresia humilis* (C. A. Mey. ex Trautv.) Sergiev, *Spiraea alpine* Pall., and *Potentilla chinensis* Ser. According to the FAO World Reference Base (WRB) for Soil Resources, the primary soil type is a Cambisol under shrubs and meadows. The parent materials are mainly eluvial-slope deposits and alluvial and diluvial parent materials.

### Sample collection

Two shrubland sites and one meadow site were selected in a relatively flat and consistent soil parent material by field verification at an altitude of 3,400–3,550 m on the principle of proximity, near Langshidang Village, Jialang Valley. At the first site, the vegetation was dominated by *Rhododendron thymifolium* (RHO, 3,430 m), accompanied by *Rhododendron capitatum* Maxim., *Salix oritrepha*, and *Spiraea alpina*. The average plant height was 0.95 m, and the coverage was 45%. The main herbs were *Polygonum viviparum* L., *Eragrostis pilosa* (Linn.) Beauv., *Chrysanthemum indicum* Thunb., and *Leontopodium leontopodioides* (Willd.) Beauv. Additionally, much of the ground was covered by moss. In the second site, the vegetation was dominated by *Potentilla fruticosa* (POT, 3,470 m), accompanied by *Spiraea alpina*. The average plant height was 0.47 m, and the coverage was 40%. The main herbs were *Polygonum viviparum*, *Leontopodium leontopodioides* (Willd.) Beauv., and *Rubus corchorifolius* L. f. The third site consisted of a subalpine meadowland (MEA, 3,550 m), with the primary plant species being *Kobresia myosuroides*, *Stellaria* media, *Elymus dahuricus*, and *Potentilla chinensis*. The average plant height was 0.07 m and the coverage was 47%.

In August 2018, we selected five sample plots (50 m × 50 m) at each vegetation type site. A sub-plot (20 m × 20 m) was then randomly selected for each plot. From each sub-plot, seven soil cores 0–10 cm deep and 5 cm in diameter were randomly taken using a cylindrical corer, and all cores were pooled into a single sample. Thus, five composite samples were collected for each vegetation type. The samples were immediately sealed in plastic bags and transported to the laboratory in portable containers at 4 °C. Each sample was divided into three subsamples after removing stones, roots, and litter. For each sample, one subsample was dried for analysis of soil chemical properties, one was stored at 4 °C to analyze dissolved organic matter and enzyme activity, and the third was stored at −80 °C for microbial PLFA analysis.

### Soil physicochemical analyses

The physicochemical properties of soil were mainly determined according to the method by Zhang & Gong (2012). Soil water holding capacity (WHC) was measured gravimetrically using cutting rings. Soil pH was measured using an PHS-3E pH meter (INESA Scientific Instrument, Shanghai, China) with a 1:2.5 (m/v) soil-to-water ratio. Soil organic carbon (SOC) content was determined using the dichromate capacity method. Soil total nitrogen (TN) was determined using the Kjeldahl method. Active organic carbon (AOC) was determined using potassium permanganate (Weil et al., 2003). To measure soil dissolved organic carbon (DOC) and dissolved total nitrogen (DTN), soil samples were extracted with deionized water (a soil-to-water ratio of 1:5), and DOC and DTN were determined using a Multi N/C 3100 analyzer (Analytik Jena, Jene, Germany) (Jones & Willett, 2006). Available phosphorus (AP) was determined by colorimetric assay at 880 nm using a spectrophotometer (Purkinje General, Beijing, China) (Vogt, Tilley & Edmonds, 2015). Available potassium (AK) was extracted with ammonium acetate and analyzed using flame photometry.

### Extraction and determination of microbial PLFA

In this study, we conducted PLFA analysis to evaluate the soil microbial biochemical composition. Soil PLFAs were extracted following a previously described procedure (Kourtev, Ehrenfeld & Häggblom, 2002). Briefly, fatty acids were extracted by mixing chloroform, methanol, and phosphate buffer (50 mM, pH 7.4) in a 1:2:0.8 ratio. Soil samples were extracted using deionized water (1:2.5, fresh soil/extraction reagent) and then subjected to oscillating centrifugation. The polar lipids in the supernatant were extracted with methanol and freeze-dried with liquid nitrogen. Polar lipids were saponified and methylated. PLFAs were designated according to the standard PLFA nomenclature, as described previously (Zelles, 1999). The fatty acid composition was analyzed using the MIDI Sherlock MIS 4.5 system (MIDI, Newark, NJ, USA). In this study, PLFAs were extracted at the Institute of Botany, Chinese Academy of Sciences, and analyzed using the institute’s fatty acid mapping data.

In a recent study, different PLFAs were identified with gram-negative bacteria (GN), gram-positive bacteria (GP), bacteria (BAC), fungi (FUN), actinomycetes (ACT), and arbuscular mycorrhizal fungi (AMF) (Aciego Pietri & Brookes, 2009). The PLFAs associated with GP bacteria included i13:0, a13:0, i14:0, a14:0, i18:0, i15:0, a15:0, i16:0, a16:0, i17:0, a17:0, i19:0, a19:0, and i20:0. For GN bacteria, they were 14:1w5c, 15:1w6c, 16:1w9c, 16:1w7c, i17:1w9c, 17:1w8c, 16:0 2OH, 18:1w7c, 18:1w5c, and 20:1w9c (Yao et al., 2018). For general BAC, the PLFAs were 12:0, 13:0, 14:0, 15:0, 17:0, 18:0, 16:0N alcohol, 20:0, cy17:0, and cy19:0 (Yao et al., 2018; Zhang et al., 2018); for FUN, they were 18:3w6c, 18:2w6c, and 18:1w9c (Kang et al., 2018). For ACT, the PLFAs were 10Me16:0, 10Me17:0, and 10Me18:0 (McKinley, Peacock & White, 2005; Jílková, Cajthaml & Frouz, 2018); and for AMF, the PLFA was 16:1w5c. The combination of GN, GP, and general BAC represents a comprehensive set of bacterial biomarkers. The total microbial fatty acid content is expressed as the sum of all fatty acids with less than 20 carbon atoms, except for the internal standard (C19:0) (Fichtner et al., 2014). The F:B ratio was calculated by dividing the sum of fungal PLFA markers by the total of bacterial PLFA markers, and the ratio of gram-positive to gram-negative bacteria (GP:GN) was calculated by dividing the sum of gram-positive bacteria by that of gram-negative bacteria.

### Soil enzyme activities

The activities of β-glucosidase (BGL, EC 3.2.1.21), cellobiohydrolase (CBH, EC 3.2.1.91), N-acetyl-glucosaminidase (NAG, EC 3.2.1.52), and leucine aminopeptidase (LAP, EC 3.4.11.1) were determined using *p*NP-β-D-glucopyranoside, *p*NP-β-D-cellobioside, 4-*p*NP-N-acetyl-β-D-glucosaminide, and leucine-para-nitroanilide (leucine-*p*NA) as substrates, respectively (Robertson et al., 1999; Dick, 2011). The buffer for alkaline phosphatase (ALP, EC 3.1.3.1) was a borate buffer solution of pH 10.0. Tris solution with a buffer of pH 8.0 was used for leucine aminopeptidase, and sodium acetate solution of pH 5.0 for the other enzymes. The enzyme activity is expressed as the mass catalyzed, in micrograms of *p*NP or *p*NA, per gram of dry soil per hour (μg/g·h).

### Statistical analysis

The Shannon–Wiener diversity index, Simpson diversity index, and Pielou evenness index were used to calculate the diversity of soil microbial PLFAs in different analysis samples. The formulas are as follows:

$$\text{Shannon}-\text{Wiener diversity index} = {\rm - }\sum\limits_{{\rm i} = {\rm 1}}^{\rm n} {\mathop P\nolimits_{\it i} } \ln \mathop P\nolimits_{\it i} ({\it i} = 1,2,\ldots,{\it n}),$$

$$\text{Simpson diversity index} = 1 - \sum\limits_{{\it i} = 1}^{\it n} {\mathop P\nolimits_{\it i}^2 },$$

$$\text{Pielou evenness index} = {\it H}/\ln {\it S},$$

where *P*_*i*_ = N_i_/N; Ni is the content of the *i*th kind of PLFA, and N is the total PLFA content; S is the number of fatty acid types.

A one-way analysis of variance (ANOVA) followed by a least significant difference (LSD) multiple comparison test was used to compare the differences in soil physicochemical properties and soil microorganisms among the three vegetation types. Principal component analysis (PCA) was used to measure microbial biochemical composition. The Monte Carlo permutation test was used to measure soil environmental factors significantly related to the changes in microbial biochemical composition before redundancy analysis (RDA). The RDA was performed to evaluate the relationship between soil physicochemical properties and biochemical composition. These analyses were performed using SPSS 21.0 (IBM, New York, USA) and Canoco 5.0 (Microcomputer Power, Ithaca, NY, USA).

## Results

### Soil physicochemical properties

The soil physicochemical properties differed significantly among the three vegetation types (Table 1). Overall, the levels of physicochemical parameters in RHO were the highest. The soil pH of RHO was significantly higher than that of MEA and POT (*p* < 0.05), whereas no significant differences were found between POT and MEA. The WHC and content of SOC, AOC, DOC, TN, DTN, and AK in RHO were higher than that in POT and MEA. There were no significant differences in the AP content among the three study sites. The ratio of SOC to TN (C/N) for RHO was the highest, significantly higher than the C/N in MEA, whereas the C/N was not significantly different between POT and MEA.

**Table 1:** Soil physicochemical characteristics of the three vegetation types.

Parameter	RHO	POT	MEA
WHC (%)	81.90 ± 0.88 a	65.10 ± 0.76 b	54.04 ± 1.05 c
pH (H_2_O)	6.99 ± 0.05 a	6.75 ± 0.05 b	6.78 ± 0.07 b
SOC (g/kg)	92.96 ± 1.38 a	47.63 ± 1.40 b	36.90 ± 1.59 c
AOC (g/kg)	8.01 ± 0.22 a	5.81 ± 0.47 b	3.92 ± 0.76 c
DOC (mg/kg)	767.53 ± 5.89 a	662.73 ± 54.27 b	480.54 ± 9.53 c
TN (g/kg)	13.00 ± 0.20 a	8.32 ± 0.14 b	4.66 ± 0.11 c
DTN (mg/kg)	37.46 ± 3.17 a	29.66 ± 1.02 b	23.61 ± 0.85 c
AP (mg/kg)	64.89 ± 1.64 a	57.48 ± 3.28 a	59.64 ± 3.43 a
AK (mg/kg)	208.00 ± 3.74 a	192.82 ± 3.28 b	108.45 ± 2.75 c
C/N ratio	11.19 ± 1.65 a	8.66 ± 0.67 ab	6.57 ± 0.60 b

**Notes:**

The values are expressed as mean ± standard error (*n* = 5).

RHO, *Rhododendron thymifolium* Maxim. shrubland; POT, *Potentilla fruticosa* L. shrubland; MEA: *Kobresia myosuroides* meadowland; WHC: water holding capacity; SOC, soil organic carbon; AOC, active organic carbon; DOC, dissolved organic carbon; TN, total nitrogen; DTN, dissolved total nitrogen; AP, available phosphorus; AK: available potassium; C/N, ratio of SOC to TN.

Significant differences (*p* < 0.05) among the three vegetation types are based on the one-way ANOVA analysis, followed by an LSD test. Same letters indicate a lack of significant difference (a, b, c).

### PLFAs of soil microorganisms in the three vegetation types

The abundance of various PLFAs of soil microorganisms differed among the three vegetation types (Table 2). Overall, the abundance of PLFAs was the highest in RHO. The abundance of total PLFAs, GP, GN, BAC, and FUN in RHO was significantly higher than that in POT and MEA, whereas the abundance of these PLFAs in POT was higher than that in MEA. The abundance of ACT and AMF in RHO was significantly higher than that in POT and MEA, whereas there was no significant difference between POT and MEA. The F:B ratio in RHO and POT was significantly higher than in that MEA. There was no significant difference in the GP:GN ratios among the three vegetation types.

**Table 2:** Abundances of PLFAs of different soil microbial groups in the three vegetation types.

Microbial community	RHO	POT	MEA
totPLFAs (nmol/g)	55.16 ± 0.39 a	46.88 ± 1.98 b	38.60 ± 1.16 c
GP (nmol/g)	10.91 ± 0.27 a	8.49 ± 0.04 b	6.63 ± 0.38 c
GN (nmol/g)	15.39 ± 0.43 a	11.70 ± 0.30 b	8.72 ± 0.32 c
BAC (nmol/g)	45.10 ± 0.77 a	30.67 ± 0.46 b	24.48 ± 0.72 c
FUN (nmol/g)	4.09 ± 0.05 a	3.19 ± 0.08 b	1.51 ± 0.17 c
ACT (nmol/g)	4.28 ± 0.13 a	2.31 ± 0.13 b	2.34 ± 0.22 b
AMF (nmol/g)	1.94 ± 0.19 a	1.24 ± 0.11 b	1.23 ± 0.21 b
F:B	0.09 ± 0.00 a	0.10 ± 0.01 a	0.07 ± 0.01 b
GP:GN	0.76 ± 0.02 a	0.73 ± 0.02 a	0.71 ± 0.01 a

**Notes:**

totPLFAs, total phospholipid fatty acids; GP, gram-positive bacteria; GN, gram-negative bacteria; BAC, bacteria; FUN, fungi; ACT, actinobacteria; AMF, arbuscular mycorrhizal fungi; F:B, the ratio of FUN to BAC; GP:GN, the ratio of GP to GN.

Significant differences (*p* < 0.05) among the three vegetation types are based on one-way analysis of variance (ANOVA) followed by an LSD test. Same letters indicate a lack of significant difference (a, b, c).

### Diversity of soil PLFAs

The Shannon–Wiener index and the Pielou evenness index of soil PLFAs in RHO were the highest among the three vegetation types (Table 3). According to the one-way ANOVA, the Simpson index was significantly lower in RHO than in the other two vegetation types.

**Table 3:** Diversity of soil PLFAs in the three vegetation types.

Parameter	RHO	POT	MEA
Shannon–Wiener index	1.34 ± 0.05 a	1.28 ± 0.15 a	1.28 ± 0.04 a
Simpson index	0.32 ± 0.03 b	0.49 ± 0.06 a	0.50 ± 0.04 a
Pielou evenness index	0.72 ± 0.03 a	0.71 ± 0.08 a	0.75 ± 0.02 a

**Notes:**

Different letters indicate significant differences (*p* < 0.05) among the three vegetation types based on the one-way ANOVA analysis, and followed by an LSD test.

### Soil enzyme activities

The soil enzyme activities differed among the three vegetation types (Table 4). Overall, the soil enzyme activity was the highest in RHO. The activities of BGL, CBH, and LAP were the highest in RHO among the three vegetation types, followed by POT and MEA. The activities of NAG and ALP were the highest in RHO, whereas no significant difference between POT and MEA was found.

**Table 4:** Activities of soil enzymes in the three vegetation types.

Enzyme	RHO	POT	MEA
BGL (μg/g·h)	192.65 ± 25.6 a	128.50 ± 16.85 b	73.36 ± 4.10 c
CBH (μg/g·h)	42.39 ± 3.48 a	31.06 ± 3.43 b	20.83 ± 1.18 c
NAG (μg/g·h)	79.95 ± 13.52 a	41.00 ± 8.48 b	19.09 ± 2.17 b
LAP (μg/g·h)	200.30 ± 29.46 a	128.81 ± 11.20 b	70.44 ± 9.04 c
ALP (μg/g·h)	1,251.82 ± 153.35 a	689.95 ± 102.96 b	558.54 ± 78.05 b

**Notes:**

BGL, β-glucosidase; CBH, cellobiohydrolase; NAG, N-acetyl-glucosaminidase; LAP, leucine aminopeptidase; ALP, alkaline phosphatase.

Significant differences (*p* < 0.05) among the three vegetation types are based on the one-way ANOVA followed by LSD test. Same letters indicate a lack of significant difference (a, b, c).

### PCA of soil microbial biochemical composition

**Figure 1:**
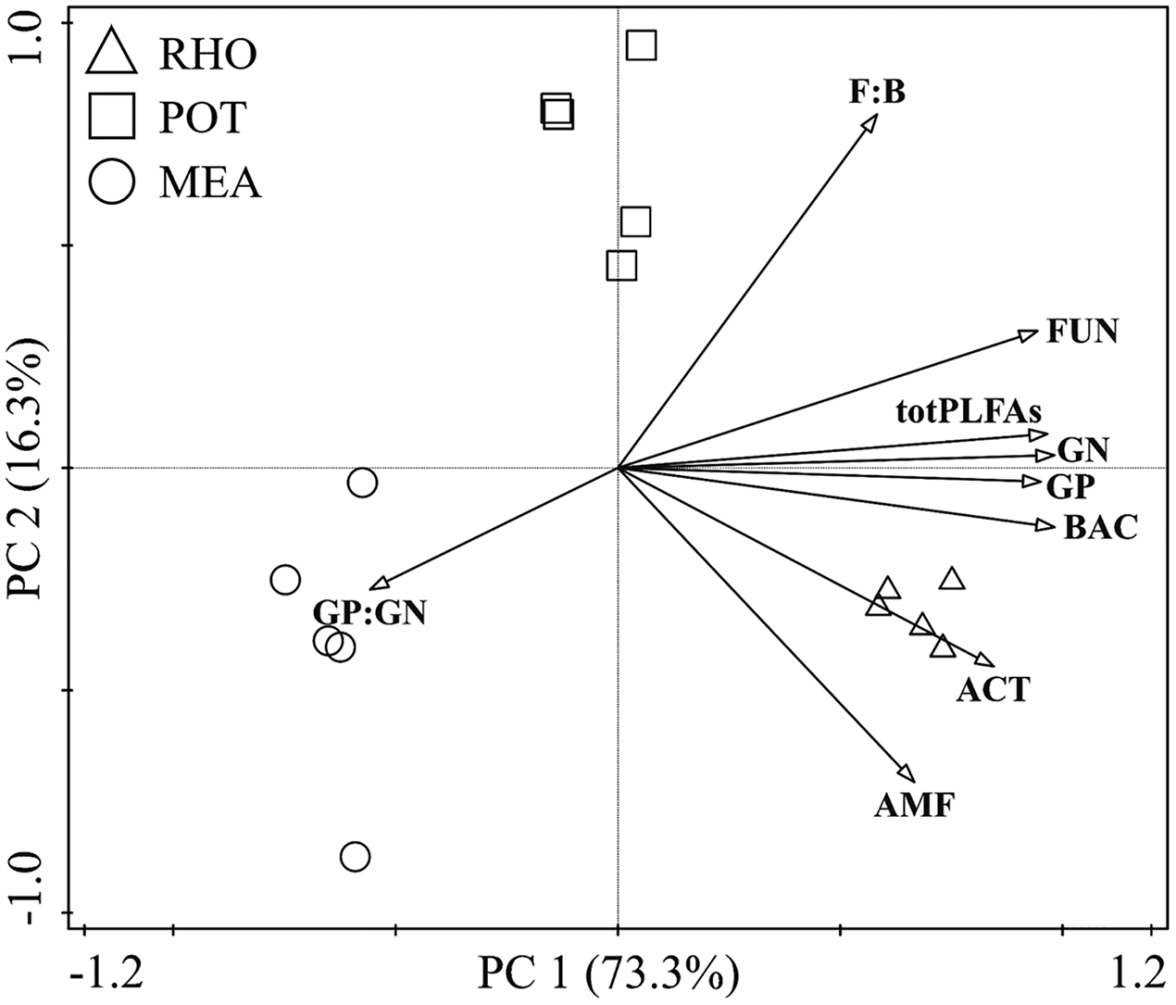
Principal component analysis of soil microbial community in the three vegetation types.

The PCA showed that the biochemical composition of the different vegetation types was well separated (Fig. 1). RHO and MEA showed no separation in the second principal component (PC2), whereas they were separated from POT. However, POT and MEA were separated from RHO in the first principal component (PC1). PC1 explained 73.3% of the variance in the dataset, whereas only 16.3% of the variance was explained by PC2. All PLFAs were positively correlated with PC1, except for GP:GN. We found that PC2 was positively driven by F:B, FUN, totPLFAs, and GN, whereas it was negatively driven by GP, BAC, ACT, AMF, and GP:GN.

### Relationships among soil physicochemical properties, microbial community, and enzyme activities

**Figure 2:**
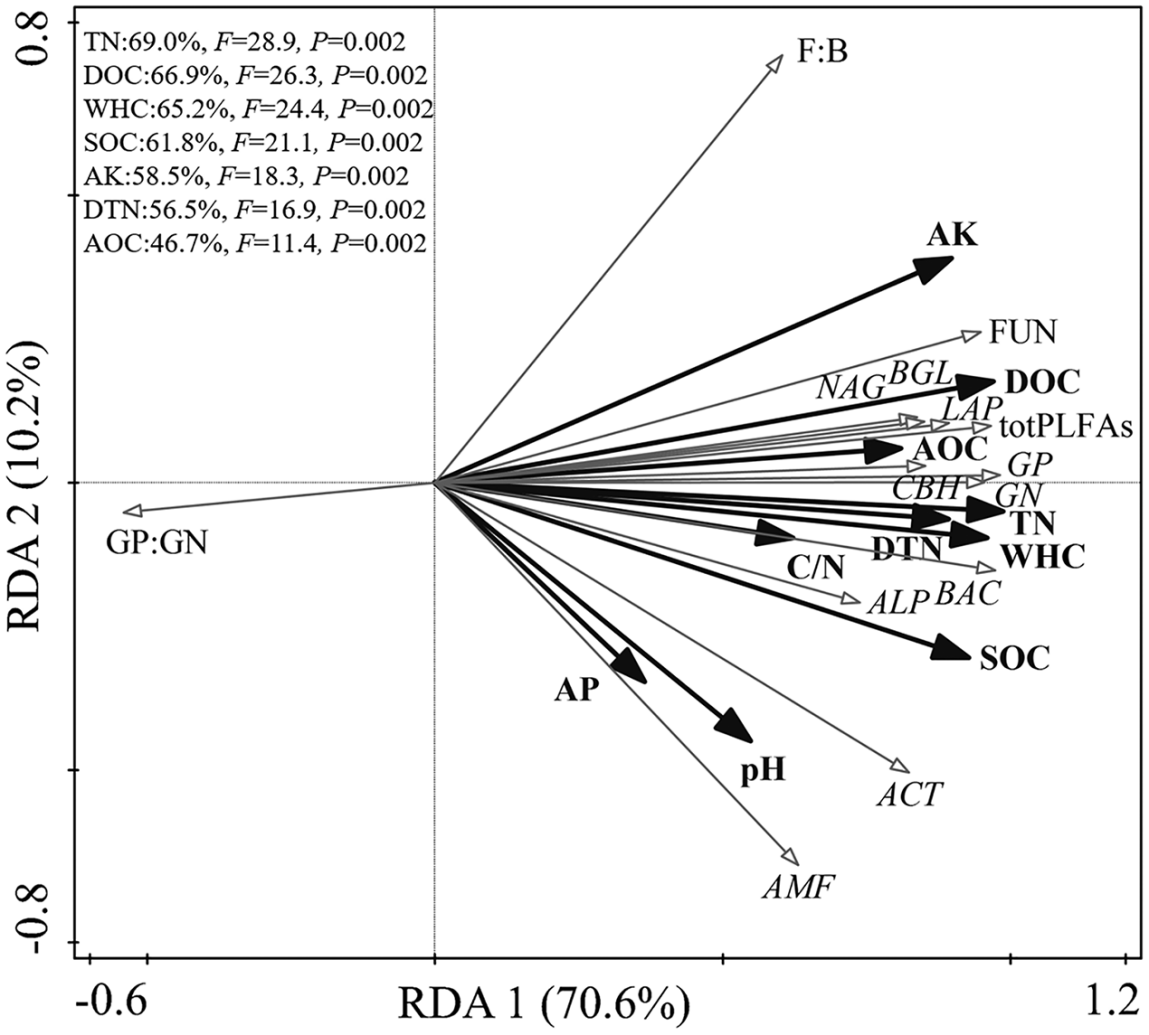
Redundancy analysis of the relationship among soil physicochemical properties, soil microbial community, and enzyme activities.

The RDA biplot showed the physicochemical properties that primarily contributed to the microbial biochemical composition and function (Fig. 2). The first axis explained 69.9% of the total variance, and the second explained 10.2%. The WHC, TN, DOC, SOC, AK, DTN, and AOC content were the main factors affecting the microbial biochemical composition and enzyme activities. Specifically, TN, DOC, WHC, and SOC could explain 69.0%, 66.9%, 65.2%, and 61.8% of the total variance, respectively.

The correlation analysis results of soil physicochemical properties, soil microbial biochemical composition, and enzyme activities are shown in Table 5. Soil pH was positively correlated with GP, GN, BAC, ACT, and AMF. The WHC, and content of soil SOC, TN, DOC, AOC, DTN, AK, and C/N showed significant positive correlations with total PLFAs, GP, GN, BAC, FUN, and ACT. The GP:GN showed significant negative correlations with the content of TN, DOC, and AK. All enzyme activities showed significant positive correlations with the WHC, SOC, TN, DOC, AOC, DTN, and AK contents. The CBH and ALP activities were positively correlated with pH and C/N.

**Table 5:** Correlations among soil physicochemical properties, microbial community, and enzyme activities.

Parameter	WHC	pH	SOC	AOC	DOC	TN	DTN	AP	AK	C/N
totPLFAs	0.946**	0.504	0.904**	0.854**	0.956**	0.974**	0.867**	0.354	0.886**	0.583*
GP	0.975**	0.628*	0.929**	0.777**	0.933**	0.949**	0.831**	0.482	0.848**	0.560*
GN	0.920**	0.567*	0.934**	0.777**	0.941**	0.968**	0.831**	0.449	0.868**	0.527*
BAC	0.943**	0.652**	0.978**	0.791**	0.905**	0.978**	0.890**	0.410	0.679**	0.674**
FUN	0.941**	0.495	0.859**	0.835**	0.979**	0.961**	0.883**	0.254	0.955**	0.620*
ACT	0.874**	0.677**	0.924**	0.618*	0.727**	0.860**	0.811*	0.381	0.568*	0.614*
AMF	0.706**	0.679**	0.824**	0.589*	0.510	0.684**	0.643**	0.454	0.323	0.358
F:B	0.533*	0.022	0.342	0.553*	0.719**	0.570*	0.504	−0.060	0.894**	0.348
GP:GN	−0.585*	0.001	−0.472	−0.396	−0.524*	-0.573*	−0.456	−0.008	-0.558*	−0.183
BGL	0.770**	0.510	0.770**	0.665**	0.802**	0.809**	0.740**	0.431	0.715**	0.382
CBH	0.805**	0.639*	0.743**	0.646**	0.786**	0.834**	0.857**	−0.015	0.712**	0.695**
NAG	0.721**	0.318	0.749**	0.626*	0.782**	0.773**	0.678**	0.414	0.689**	0.478
LAP	0.781**	0.388	0.776**	0.589*	0.818**	0.808**	0.677**	0.394	0.739**	0.402
ALP	0.713**	0.524*	0.772**	0.570*	0.706**	0.750**	0.697**	0.426	0.568*	0.673**

**Notes:**

* Significant correlation (*p* < 0.05).

** Significant correlations (*p* < 0.01).

## Discussion

### Effect of vegetation types on the soil properties

Vegetation types substantially change soil properties *via* the transformation of organic substances (Lu et al., 2014). Moreover, the nature of the change is related to plant features such as size, cover, and root system. Shrublands are more effective than meadows in augmenting the organic carbon and nutrient content by increasing litter biomass and improving the microenvironment (Drewnik et al., 2016). In general, larger shrubs have higher soil nutrient status (Yao et al., 2019). In our study, the content of SOC, DOC, TN, and TDN in shrublands was higher than that in the meadow. The results concur with those of a previous study (Qi et al., 2015), which reported a higher content of SOC and TN in shrubland than in grassland. In this study, we found that the DTN content in RHO was higher than that in POT; this difference was related to the lower aboveground biomass in POT (Peng et al., 2018). Additionally, the moss cover in RHO may increase the carbon and nitrogen content. This suggestion is supported by the findings of a previous study (Sun et al., 2017), which reported that moss-covered land could present increased SOC, DOC, and TN content.

### Effect of vegetation type on the soil microbial community and its function

Based on their characteristics, different vegetation types may change soil environmental conditions, further affecting the growth, reproduction, and community structure of soil microorganisms (Zheng et al., 2019). Meadows have a lower biomass, shallower root distribution, and lower soil microbial properties than shrubs. A previous study showed that shrublands tend to have a higher total quantity of soil microorganisms and a greater abundance of fungi than meadows (Li et al., 2017). Higher GP abundance has been found under shrublands than under grasslands in rocky desertified regions (Zhao et al., 2019). Moreover, the F:B ratio reflects the soil ecological function under the different vegetation types, and a higher F:B ratio indicates a higher soil carbon content and greater carbon use efficiency. In our study, shrublands presented a higher F:B ratio than meadows, consistent with the results of a previous study (Djukic et al., 2010). This result indicated that more litter-derived carbon under shrubland, with higher aboveground biomass and root capacity, can be transformed to SOC, which is considered a crucial growth substrate for soil microorganisms (Li et al., 2009) and can further increase the soil microbial biomass (Soares & Rousk, 2019). The abundance of total PLFAs, GP, GN, BAC, and FUN under shrubs was higher than that under meadows in our study. The result was consistent with the variation of F:B ratios between the shrublands and the meadow.

Different plant species within the same vegetation type can also significantly affect the composition of soil microorganisms owing to their different growth characteristics (Massenssini et al., 2015). In this study, the abundance of all PLFAs in RHO was higher than that in POT. These results may be related to the higher aboveground biomass under RHO than under POT (Nie et al., 2018). In addition, the development of moss in RHO may increase the soil microbial biomass, which is supported by a previous study (Shen et al., 2018) that reported higher microbial biomass under moss cover.

Vegetation types may influence the activity of soil enzymes by affecting the metabolic activities of the microorganisms (Caldwell, 2005). For example, the BGL and ALP activity in shrubland was lower than that in grassland (Jin et al., 2019). The opposite effects were observed in another study (Cui et al., 2019), which reported that shrubs might increase soil enzyme activities. This means that the variations in soil enzymes are specific and related to the growing conditions of the vegetation. Our results suggest that shrubland performs better in terms of BGL, CBH, and LAP activity than meadow. This finding is supported by those of a previous study (Liu et al., 2021). In addition, RHO had better soil enzyme activity than POT, which was consistent with the results of Hu et al. (2016). We observed higher soil enzyme activities in shrubland partially explaining the higher microbial abundance in this vegetation type. Moreover, this supposition is supported by the fact that enzyme activities were significantly correlated with total PLFAs, FUN, and BAC (Fig. 2).

### Soil properties driving the soil microbial community and its function

The development of microorganisms requires sufficient moisture to meet the microbes’ physiological needs. The significant positive correlation between WHC, microbial biomass, and enzyme activity was consistent with previous studies (Cui et al., 2019; Kang et al., 2018). As a source of energy and nutrients for microorganisms, soil organic matter affects the growth and development of microorganisms, further influencing soil microorganisms’ biochemical composition and enzyme activities (Li et al., 2016). The AOC and DOC are considered important labile components, and the increase in labile organic matter can provide an adequate and appropriate decomposition environment for microorganisms, further driving the shift in microbial community composition (Malik et al., 2016). In the three vegetation types, the in total PLFAs, GP, GN, FUN, and BAC abundance, as well as in soil enzyme activities were strongly driven by DOC, SOC, and AOC. In addition, DOC and SOC accounted for more than 60% of the total variance (Fig. 2). These results are consistent with those of a previous study (Xiang et al., 2019; Xu et al., 2020).

Nitrogen has a significant influence on soil microbial biomass. In addition, increasing the nitrogen content can accelerate microorganism reproduction and change the abundance composition of AMF (Lv et al., 2017; Yokobe, Hyodo & Tokuchi, 2018). The above perspective indicates that the TN and TDN contents were largely responsible for the variations in soil microorganism species in our study. Therefore, higher nitrogen content can improve the availability of the nutrient source, thus boosting soil microorganisms (Kong et al., 2011). As the soil physicochemical properties were studied, the influence of vegetation character on soil microorganisms and enzymes in subalpine zones should be studied further.

## Conclusions

In the high-altitude areas of the Qilian Mountains, the biochemical composition of soil microorganisms and the enzyme activities of soil microbial communities under two shrublands and one meadow were investigated. The results showed that the shrublands had a greater abundance of total PLFAs, GP, GN, BAC, and FUN than the meadow. In addition, the biochemical composition of the soil microbial community was considerably affected by the carbon and nitrogen content. Higher microorganism abundance and higher F:B ratio in the shrubland than in the meadow suggested that shrubs can improve the soil physicochemical properties and the microbial biochemical composition. Moreover, the activity of soil enzymes involved in the decomposition of organic carbon and nitrogen was higher under the shrubs than under the meadow. Furthermore, RHO shrubland had higher microbial biomass and enzyme activity than POT shrubland. The RDA indicated that the biochemical composition of the soil microorganisms and enzyme activities were driven by SOC, TN, DOC, WHC, SOC, AK, and DTN. Thus, the shrublands with higher biomass can improve soil moisture status and increase soil carbon and nitrogen content, especially active carbon, active nitrogen, and further increase the abundance of total PLFAs, bacteria, and fungi. The increase of microbial biomass indirectly enhances the activity of relevant soil enzymes. Overall, our findings showed that the variations of PLFA abundance and enzyme activities could be attributed to shrub species, especially the evergreen shrubs that can create more favorable development conditions for the soil microorganisms in vulnerable subalpine regions. The results of this study provide a theoretical basis for investigating the soil biogeochemical cycle process and a scientific basis for soil management and vegetation restoration in the subalpine regions.

## Supplemental Information

10.7717/peerj.13188/supp-1 Supplemental Information 1 Soil physicochemical properties.

10.7717/peerj.13188/supp-2 Supplemental Information 2 Phospholipid fatty acids and enzyme activities.

## Additional Information and Declarations

### Data Availability

The following information was supplied regarding data availability: The raw measurements are available in the Supplemental Files.
